# Androgen receptor is a potential novel prognostic marker and oncogenic target in osteosarcoma with dependence on CDK11

**DOI:** 10.1038/srep43941

**Published:** 2017-03-06

**Authors:** Yunfei Liao, Slim Sassi, Stefan Halvorsen, Yong Feng, Jacson Shen, Yan Gao, Gregory Cote, Edwin Choy, David Harmon, Henry Mankin, Francis Hornicek, Zhenfeng Duan

**Affiliations:** 1 Sarcoma Biology Laboratory, Department of Orthopaedic Surgery, Massachusetts General Hospital and Harvard Medical School, 55 Fruit Street, Jackson 1115, Boston, Massachusetts 02114USA; 2 Department of Endocrinology, Wuhan Union Hospital, Tongji Medical College, Huazhong University of Science and Technology, 1277 Jie Fang Avenue, Wuhan, 430022, China; 3 Center for Computational and Integrative Biology (CCIB), Massachusetts General Hospital, Boston, Massachusetts 02139USA; 4 Department of Orthopaedic Surgery, Wuhan Union Hospital, Tongji Medical College, Huazhong University of Science and Technology, 1277 Jie Fang Avenue, Wuhan, 430022, China; 5 Division of Hematology and Oncology, Massachusetts General Hospital and Harvard Medical School, Boston, Massachusetts 02114, USA

## Abstract

Osteosarcoma is the most common bone cancer in children and adolescents. Previously, we have found that cyclin-dependent kinase 11 (CDK11) signaling was essential for osteosarcoma cell growth and survival. Subsequently, CDK11 siRNA gene targeting, expression profiling, and network reconstruction of differentially expressed genes were performed between CDK11 knock down and wild type osteosarcoma cells. Reconstructed network of the differentially expressed genes pointed to the AR as key to CDK11 signaling in osteosarcoma. CDK11 increased transcriptional activation of AR gene in osteosarcoma cell lines. AR protein was highly expressed in various osteosarcoma cell lines and patient tumor tissues. Tissue microarray analysis showed that the disease-free survival rate for patients with high-expression of AR was significantly shorter than for patients with low-expression of AR. In addition, AR gene expression knockdown via siRNA greatly inhibited cell growth and viability. Similar results were found in osteosarcoma cells treated with AR inhibitor. These findings suggest that CDK11 is involved in the regulation of AR pathway and AR can be a potential novel prognostic marker and therapeutic target for osteosarcoma treatment.

Osteosarcoma is the most common primary malignant bone cancer. It is also the most common bone cancer in children and young adults^1^. Current treatment for osteosarcoma is effective but can be limited. Surgery is important and chemotherapy significantly improves the prognosis for many osteosarcoma patients. However, despite aggressive chemotherapy, more than 30% of patients with localized osteosarcoma experience metastatic disease; survival after metastases is less than one year^2^. Thus, more effective therapeutic strategies are required for the treatment of osteosarcoma.

Cyclin-dependent kinases (CDKs) are the family of serine/threonine protein kinases and control progression through the cell cycle. Among cell cycle proteins CDKs are essential. High activation and expression of CDKs have been found in different types of cancer, and are becoming popular and potentially useful therapeutic targets^3^,^4^,^5^. Recently, the FDA has approved the CDK4/6 inhibitor, palbociclib, for the treatment of metastatic breast cancer^6^. CDK11, also named as PITSLRE or CDC2L1, is a serine/threonine protein kinase in the CDK family. CDK11 seem to have multiple roles in cell cycle progression, RNA splicing and transcriptional regulation. CDK11 expression is ubiquitous and constant throughout the cell cycle^7^,^8^. There are three major isoforms of CDK11 proteins, CDK11p110, CDK11p58, and CDK11p46^9^. The p110 isoform of CDK11 is expressed in all examined human cancer cell lines, including breast cancer, multiple myeloma, osteosarcoma, liposarcoma and other types of human cancers^9^,^10^,^11^,^12^. However, CDK11p58 protein is specifically translated from an internal ribosome entry site and expressed only in the G2/M phase of the cell cycle. Recent studies have shown that CDK11 also plays critical roles in cancer cell growth and proliferation^10^,^12^,^13^,^14^. Previously, we have demonstrated that CDK11 expression is essential in osteosarcoma cell growth and survival^10^,^15^. Osteosarcoma cells display high-expression of CDK11. CDK11 expression knock-down by either lentivirus delivered shRNA or transfected siRNA inhibits cell growth and induces apoptosis in osteosarcoma cells^10^. Knockout of CDK11 by CRISPR-Cas9 has also similar effects^15^. Immunohistochemical analysis showed that osteosarcoma patients with high-expression of CDK11 in tumors had significantly shorter survival than those with low-expression^10^. Therefore, developing strategies targeting the CDK11 pathway may provide therapeutic benefit for the treatment of osteosarcoma. Currently, there is no available inhibitor of CDK11. In addition, the biological network of CDK11 signaling in osteosarcoma is poorly understood.

In this study, CDK11 siRNA targeting, gene expression profiling, and gene network reconstruction of differentially expressed genes were performed to investigate the difference in signaling between CDK11 knockdown and wild type in osteosarcoma cells. In an effort to validate and further understand the AR connection, we experimentally examined the expression and relationship between CDK11 and AR in osteosarcoma cell lines and patient tissues. The functional roles of AR in growth/viability and migration of osteosarcoma cell lines were evaluated. Furthermore, we determined the effects of AR specific inhibitor bicalutamide (also known as casodex) on osteosarcoma cells and interrogated the underlying mechanism of CDK11 and AR gene network in osteosarcoma.

## Materials and Methods

### Cell lines and cell culture

The human osteosarcoma cell lines, U-2OS (origin from a female 15y old osteosarcoma patient), SaOS, MNNG/HOS, and MG63 were purchased from the American Type Culture Collection (Rockville, MD) in 2014. The human osteosarcoma KHOS (origin from a female 13y old osteosarcoma patient) cell line was provided by Dr. Efstathios Gonos (Institute of Biological Research & Biotechnology, Athens, Greece) in 2008. KHOS and U-2OS are two commonly used cell lines in osteosarcoma research. The human osteoblast cell lines NHOst and HOB-c were purchased from LonzaWalkersville (Walkersville, MD) and PromoCell GmbH (Heidelberg, Germany) respectively in 2013. The osteosarcoma cell lines were cultured in RPMI-1640 (Life Technologies, Grand Island, NY) supplemented with 10% fetal bovine serum, 100 U/mL penicillin, and 100 mg/mL streptomycin (Life Technologies, Carlsbad, CA). The osteoblast cell lines were cultured in osteoblast growth medium (PomoCell) with 10% fetal bovine serum. All cells were maintained in a humidified atmosphere containing 5% CO_2_ at 37 °C. Bicalutamide (Sigma-Aldrich, St. Louis, MO) is a synthetic, non-steroidal AR antagonist and a pure antiandrogen used in the treatment of prostate cancer, hirsutism, and other androgen-dependent conditions. Doxorubicin was purchased from APP pharmaceuticals, LLC, (Lake Zurich, Illinois).

### CDK11 siRNA knockdown and RNA extraction for gene profiling

CDK11 knockdown in osteosarcoma cell lines was carried out with human CDK11 siRNA purchased from Life Technologies (Carlsbad, CA). The siRNA sequence targeting CDK11 corresponded to the coding region (5′-AGAUCUACAUCGUGAUGAAtt-3′) of the CDK11 gene. Nonspecific siRNA oligonucleotides (Life Technologies) were used as controls. The siRNA oligonucleotides were dissolved in nuclease-free water at a concentration of 100 mM. Osteosarcoma KHOS or U-2OS cells were transfected with Lipofectamine RNAiMax reagent (Life Technologies) according to the manufacturer’s recommendations. Total RNA was collected from these cells using TRIzol Reagent (Life Technologies) 48 hours after CDK11 siRNA transfection.

### Transcriptional profiling and array analysis

CDK11 knockdown in osteosarcoma cell lines was carried out with human CDK11 siRNA. Total RNA was processed and hybridized to Affymetrix Genechip U133 Plus 2.0 arrays (Affymetrix, Santa Clara, CA) by the Gene Array Technology Center at Harvard Medical School This array exhibits coverage of the human transcriptome with over 47,000 transcripts. We normalized the microarrays using the gcrma package from the R Bioconductor suite^16^ and extracted the generated expression values from the resultant ExpressionSet object. Next, we used a Python script to loop through each CEL file and generate the Present/Marginal/Absent calls using the mas5calls function within the affy package^17^. The mas5calls function generates the P/M/A calls according to the Wilcoxon signed rank-based gene expression presence/absence detection algorithm. After generating the P/M/A calls, we eliminated any probe absent from all CEL files, and any probe which did not have a max value above log_2_ (100). These normalized and filtered values were taken forward into the differential expression analysis. Raw microarray data has been deposited in GEO database (GEO accession number: GSE73422).

### Differential Expression Analysis

**Figure 1:**
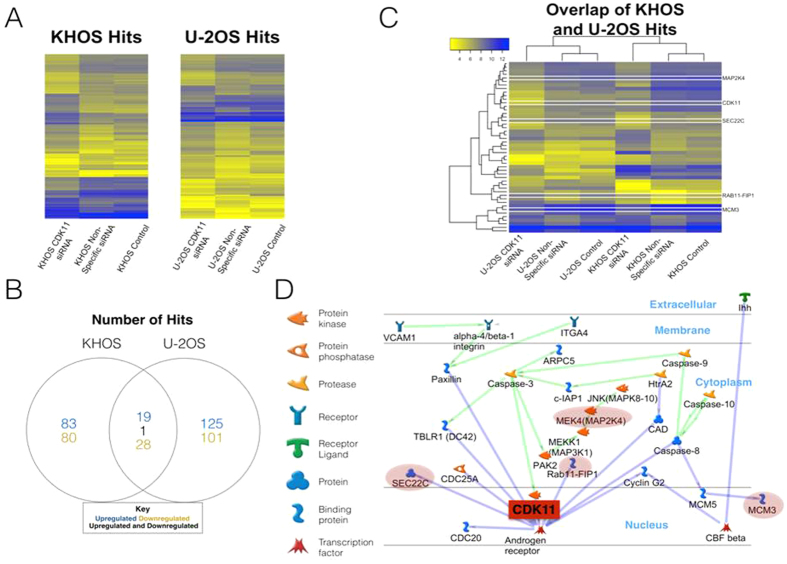
Differential gene expression in KHOS and U-2OS when CDK11 is knocked down and identification of CDK11 and Androgen Receptor gene network as central in osteosarcoma. (**A**) Heatmap of gene probes significantly perturbed in KHOS and U-2OS upon CDK11 knockdown. (**B**) Venn diagram showing the gene number breakdown of differentially expressed genes. (**C**) Heatmap and hierarchal clustering of the common differentially expressed genes in both KHOS and U-2OS. (**D**) Gene network reconstruction utilizing GeneGo. The common differentially expressed genes were used to seed network reconstruction. Two gene networks, both containing CDK11, were merged (green and blue) and trimmed for irrelevant nodes. The genes highlighted in a red shadow were perturbed upon CDK11 knockdown. The Androgen Receptor (AR) is highlighted as a central node and potentially a significant player.

We generated a dataset of expression differences (henceforth named Diff_Data) by subtracting the expression values of the CDK11 siRNA sample from the values of the non-specific siRNA sample. The starting expression data did not appear to follow a normal distribution, but Diff_Data exhibits a much better approximation of a normal distribution. We calculated the mean and standard deviation of Diff_Data and calculated z-scores for each point in Diff_Data. With a standard metric to compare probes, we extracted all probes with a score above or below 3 or −3, respectively. We then generated an overlapping hit list by taking the intersection of the lists from KHOS and U2OS. In the event of multiple probes matching the same gene, their expression values were averaged. For visualization of the data, we generated heatmaps showing the expression values of the hits using the programming language R (Fig. 1). The gene HELLS is notable in that it is the only hit list gene whose expression value did not change in the same direction between KHOS and U2OS upon transfection with the CDK11 siRNA; in KHOS it was down-regulated, while in U2OS it was up-regulated.

### Identification of CDK11 associated gene network

The common differentially expressed genes in both KHOS and U-2OS upon CDK11 knockdown were used as seeds to construct gene networks using GeneGO. Two of the constructed networks included CDK11. Both of these networks were combined and filtered for genes expressed in bone and neoplasms. The resulting network was further trimmed for irrelevant members. The final network pointed to AR as an important node (Fig. 1).

### Osteosarcoma tissue microarray (TMA) and immunohistochemical staining

Osteosarcoma TMA were generated as described previously^18^. Osteosarcoma tissue samples were obtained from the Massachusetts General Hospital Sarcoma Tissue Bank and were used in accordance with the policies of the institutional review board of the hospital (IRB protocol # 2007P-002464). The detailed sample and clinical information of the osteosarcoma TMA used in this study have been described in our previous studies^18^,^19^. CDK11 and AR expressions were evaluated by immunohistochemistry as previously described^20^. Primary AR antibody (1:50 dilution, Dako, CA) and CDK11 antibody (1:50 dilution, Cell Signaling Technology) were incubated with the TMA at 4 °C overnight in a humidified chamber. Expressions of AR and CDK11 were evaluated according to nuclear staining and calculated by the percentage of positive cells. Thereby, staining patterns were categorized into 6 groups: 0, no nuclear staining; A, 1+, <10% of cells stained positive; B, 2+, 10% to 25% positive cells; C, 3+, 26% to 50% positive cells; D, 4+, 51% to 75% positive cells; E, 5+, >75% positive cells. Cases labeled as either metastasis or recurrence refers to the disease state at the time of tumor resection/biopsy.

### CDK11 and AR siRNA transfections

CDK11 or AR knockdown in osteosarcoma cell lines was conducted by transfection of human CDK11 siRNA (Life Technologies) or AR siRNA (Sigma-Aldrich, St. Louis, MO, SASI_Hs01_00224483). Transfection of siRNA was performed using Lipofectamine RNAiMax (Invitrogen) according to the manufacturer’s instructions. Nonspecific siRNA oligonucleotide (Life Technologies) was used as a negative control.

### Western blotting assay

Osteosarcoma cells were lysed with RIPA Lysis Buffer (Upstate Biotechnology, Charlottesville, VA) plus complete protease inhibitor cocktail tablets (Roche Applied Science, IN). The components of RIPA lysis include buffer 50 mM Tris-HCl, pH7.4, 150 mM NaCl, 0.25% deoxycholic acid, 1% NP-40 and 1 mM EDTA. Protein concentrations were measured using the Bio-Rad Protein Assay kit (Bio-Rad, CA). Antibodies against AR (1:1000 dilution), CDK11 (1:1000 dilution), and β-actin (1:2000 dilution) were purchased from Cell Signaling Technology, Santa Cruz, and Sigma-Aldrich, respectively. Secondary antibodies IRDye^®^ 800CW or IRDye^®^ 680LT were purchased from LI-COR (Biosciences, NE). Western blot analysis was performed as previously reported^21^.

### Androgen Receptor transcription luciferase reporter assay

AR promoter luciferase reporter assay was performed according to the manufacturer’s protocol (LightSwitch Assay from Switch Gear Genomics, CAT^#^LS010). The reporter construct relies on RenSP luciferase driven by the AR promoter. Wild type CDK11 in pCMV6 vector (RC216465) was purchased from Origene (Rockville, MD). pEGFP-N3 plasmid was purchased from Clontech (Mountain View, CA) and served as control. KHOS and U-2OS cell lines (3*10^4^ cells per well) were seeded in 96-well plates. One day post seeding, cells were cotransfected with CDK11 plasmid (20 ng or 40 ng) and GoClone AR reporter (50 ng, Switch Gear Genomics, Prod ID S714892). To test the effect of CDK11 knockdown, KHOS and U-2OS cells were transfected with CDK11 siRNA (10 nM or 20 nM) during cell seeding. Twenty-four hours later, GoClone reporter carrying wild type AR promoter (50 ng,) was transfected in those cells. Luciferase activity with LightSwitch Assay Reagents was measured with a plate luminometer 48 hours after reporter transfection.

### MTT cell viability assay

Effects of AR siRNA or AR inhibitor bicalutamide on osteosarcoma or osteoblast cell growth and viability were assessed *in vitro* using the MTT assay. KHOS or U-2OS cell lines were transfected with AR siRNA in increasing concentrations up to 80 nM. Meanwhile, some osteosarcoma cells were dosed with bicalutamide in increasing concentration up to 100 μM. HOB-c and NHOST cells were dosed with bicalutamide in increasing concentrations up to 810 μM. 5*10^3^ cells were seeded into each well in 96-well plates. After 72 hours of incubation, 20 μL of MTT (5 mg/mL, dissolved in PBS) was added to each well and the cells were cultured for 4 hours at 37 °C. Absorbance at a wavelength of 490 nm was measured on a SPECTRAmax Microplate Spectrophotometer from Molecular Devices (Sunnyvale, CA).

### Cell Colony formation unit assay

The colony forming unit assay (CFU) assay was used to validate the effect of AR siRNA or AR inhibitor on viability and differentiation of osteosarcoma cells. KHOS or U-2OS cell lines were transfected with AR siRNA (40 nM), nonspecific siRNA (40 nM) or dosed with bicalutamide (5 μM), and DMSO (5 μM) as described above. 100 cells were seeded into each well of a 12-well plate for 7–10 days. Medium was changed every 3 days. Colonies were fixed with methanol for 10 minutes, following staining with crystal violet (0.5%) for 10 minutes. A stereo microscope and a scoring grid were used to identify and count individual colonies.

### Wound healing migration assay

In order to identify the potential role of AR in the metastasis of osteosarcoma, cell migration was assayed by multiple scratch wound assay. 1*10^5^ cells per well were plated into 12-well plates and transfected with AR siRNA (40 nM). Three parallel lines were made in confluent cell cultures with a 200 μl tip. After suspended cells were washed away with serum-free medium, the cultures were fed with complete medium again. The wounds were observed at 0, 8, and 24 hours after scratching separately, and photographed via a microscope (Nikon Instruments, Inc.) at each time point using a 10 X objective. The distance between the two edges of the wound width was quantified at 10 random sites in each image. The cell migration distance was defined as the wound width at 0 h time point minus the wound width at each time point and then divided by two.

### Three dimensional culture (3D) Growth in Suspension

Three dimensional culture (3D) growth in suspension is an improved method over 2D culture and a more accurate model to determine the behavior of cancer cell growth. KHOS and U-2OS cell lines were used and cell spheroid formation was established in HDP1096 Perfecta3D^®^ 96-Well Hanging Drop Plates (3D Biomatrix) according to the manufacturer’s protocol.

### Statistical analysis

GraphPad PRISM 5 software (GraphPad Software, Inc.) was used for statistical analyses. Results are expressed as mean ± SD and *P* values < 0.05 was regarded as statistically significant. The log-rank test was used to compare the differences in survival curves. Prognostic factors associated with overall survival or disease free survival (DFS) were investigated according to the Cox proportional hazards regression model, in a stepwise manner. Immunohistochemical staining was evaluated with the χ^2^ test to analyze the relationship between AR expression and CDK11 expression in clinical-pathological parameters of osteosarcoma.

## Results

### Identification of AR and CDK11 signaling link in osteosarcoma cell lines

CDK11 expression is known to play a crucial role in tumor cell growth and viability^10^,^21^,^22^. However, the details of CDK11 signaling network in osteosarcoma are still unknown. A better understanding of the mechanisms underlying CDK11 signaling network in osteosarcoma may allow for useful therapeutic targets. To identify the key members of the gene network associated with CDK11, we generated gene expression profiles of osteosarcoma lines when CDK11 is knocked down using Affymetrix microarrays U133 Plus 2.0 as described in the methods (GEO accession number: GSE73422). We compared gene expression profiles of CDK11 knock down in both KHOS and U2OS to the respective controls. Differential gene expression profiles, in KHOS and U2OS, produced 211 and 274 genes respectively. The two separate gene lists were compared and the common 48 genes were taken to be most significant and relevant to CDK11 gene network in osteosarcoma lines (Supplemental Table S1). These 48 genes were used as seeds to reconstruct gene networks utilizing the software suite and database GeneGo. Two of the resulting statistically significant networks included CDK11. The two networks were fused and trimmed for irrelevant members. A central node to the resulting network is the AR (Fig. 1).

### Correlation between AR and CDK11 expressions in cell lines and osteosarcoma tissues

**Figure 2:**
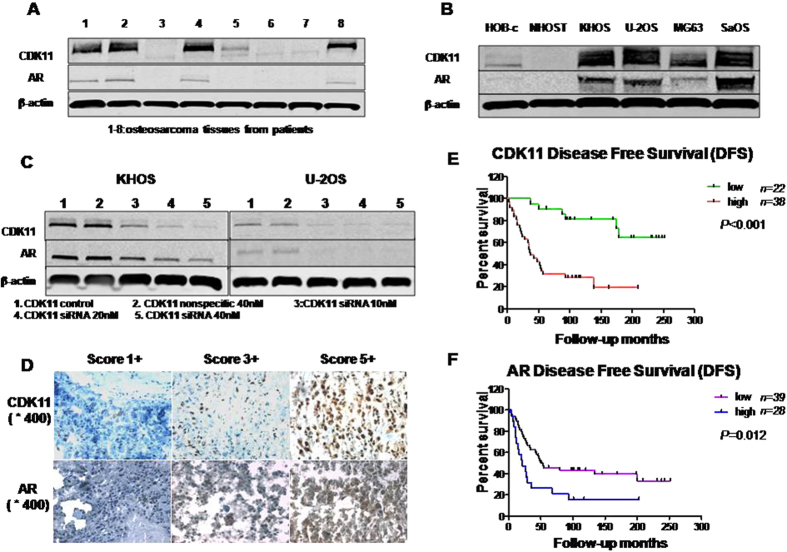
Expression of CDK11 and AR in osteosarcoma cell lines and osteosarcoma tissues. (**A**) Expressions of CDK11 and AR in osteosarcoma tissues. (**B**) Expressions of CDK11 and AR in osteosarcoma cell lines and normal osteoblast cell lines. (**C**) Expression of AR in osteosarcoma with CDK11 siRNA. (**D**) Representative images of different immunohistochemical staining intensities of AR and CDK11 are shown in osteosarcoma tissues. The percentage of cells showing positive nuclear staining for AR and CDK11 was calculated by reviewing the entire spot. On the basis of the percentage of cells with positive nuclear staining, the staining patterns were categorized into 6 groups: 0, no nuclear staining; 1, 1+, <10% of cells stained positive; 2, 2+, 10% to 25% positive cells; 3, 3+, 26% to 50% positive cells; 4, 4+, 51% to 75% positive cells; and 5, 5+, >75% positive cells (Original magnification, ×400). (**E**) Kaplan-Meier survival curve of patients with osteosarcoma were subgrouped as either CDK11 low staining (staining ≤2) or high staining (staining ≥3). (**F**) Kaplan-Meier disease free survival curve of patients with osteosarcoma were subgrouped as either AR low staining (AR staining ≤2) or high staining (AR staining ≥3).

CDK11 and AR have been shown to be involved in the regulation of cell viability and apoptosis in some human cancers^23^,^24^. However, no information is available on the relationship between CDK11 and AR. We examined the correlation between CDK11 expression and AR expression in both osteosarcoma cell lines and primary osteosarcoma patient tumor tissues. Four out of eight osteosarcoma tissue samples showed both AR and CDK11 proteins as highly expressed (Fig. 2A). Expressions of AR and CDK11 were also shown to be significantly higher in osteosarcoma cell lines compared to normal osteoblast cell lines. In normal human osteoblast cell lines (HOB-c and NHOST), AR and CDK11 expressions were extremely low and almost undetectable (Fig. 2B). Subsequently, we evaluated the expression of AR in response to increasing concentrations of CDK11 siRNA. CDK11 expression decreased gradually in response to the increasing concentrations of CDK11 siRNA (10 nM to 40 nM). AR expression showed the same trend as CDK11 in both KHOS and U-2OS cell lines (Fig. 2C). These data demonstrate a correlation between CDK11 and AR expression in osteosarcoma and suggest a regulation of AR expression by CDK11.

### Prognostic value of AR and CDK11 in osteosarcoma patients

We next examined the expression of AR in patient tumor tissue and its potential diagnostic value in clinical osteosarcoma. Among the available specimens, 87 of 114 (77.2%) exhibited immunostaining in the cell nucleus, ranging from no staining (5 of 87, 5.8%), 1+ staining (24 of 87, 27.6%), 2+ staining (22 of 87, 25.3%), 3+ staining (19 of 87, 21.8%), 4+ staining (15 of 87, 17.2%), and 5+ staining (2 of 87, 2.3%); 27 specimens were not counted due to tissue loss on the TMA slide. The median age for the patients in the TMA was 30 years (range from 6 to 72), and predominantly male (62.1% of patients). Among the available specimens, less than 25% of positive staining were classified as AR-low patients (58.6%); while intense staining (over 25%) were classified as AR-high patients (41.4%). We then compared the expression levels of AR in primary osteosarcoma specimens from cases with different responses to preoperative chemotherapy. There were significant differences in AR expression between good and poor response to preoperative chemotherapy (*P* = 0.047). Patients with high-expression of AR responded well to preoperative chemotherapy, but a with a trend towards a worse 5-year survival (Table 1). However, there were no significant relationship between AR expression and the clinical pathological features of the human tumor samples such as age at surgery, gender, tumor grade, metastasis, or the presence of local recurrence (Table 1).

We further examined the relationship between CDK11 and AR expression in the available TMA. We found a close relationship between CDK11 and AR staining patterns in cells with positive nuclei staining (Fig. 2D). The Spearman’s correlation coefficient of CDK11 and AR staining patterns in osteosarcoma TMA was 0.701 (*P* < 0.001). Kaplan-Meier analysis showed that osteosarcoma patients had a lower disease free survival in the high CDK11 or AR expressions group compared with patients in the low CDK11 or AR expressions group (*P* < 0.001 and *P* = 0.012) (Fig. 2E and F). To determine whether AR expression was an independent prognostic factor for osteosarcoma patients, the Cox regression model was adopted (Supplemental Table S2). Multivariate Cox regression analysis indicated that metastasis was significantly associated with shorter 5-year overall survival and disease-free survival (*P* = 0.013). However, AR expression was not an independent predictor for either 5-year overall survival or disease-free survival, although it showed a trend (Supplemental Table S2).

### CDK11 increases transcriptional activation of AR gene in osteosarcoma cell lines

**Figure 3:**
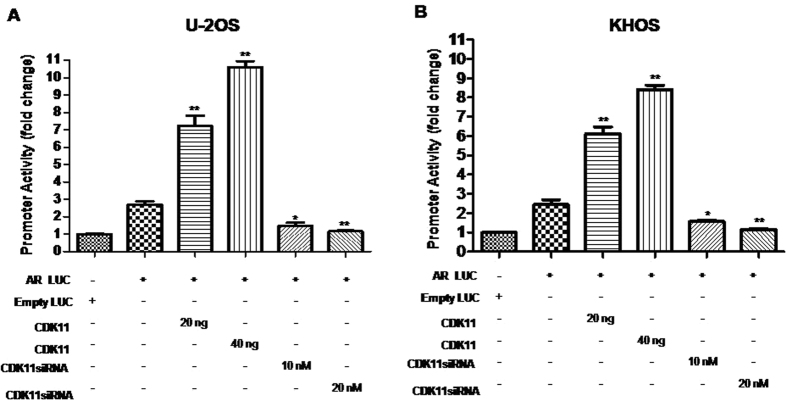
CDK11 increases transcriptional activation of the AR gene in osteosarcoma cell lines. (**A,B**) U-2OS or KHOS cells were cotransfected with CDK11 (20 ng, 40 ng) and the GoClone promoter reporter vector against the AR promoter (50 ng). CDK11 siRNA (10 nM, 20 nM) was transfected 24 hours prior to transfection with the reporter. 20 ng of an empty luciferase reporter vector was transfected to serve as a baseline, Luciferase activity was measured and normalized to the empty Luc luciferase activity. *P < 0.05, **P < 0.01 (compared with only AR LUC group).

We took advantage of a luciferase reporter assay system to further identify whether CDK11 proteins are involved in the transcriptional activity of AR. Figure 3A demonstrates that the effect of CDK11 on AR transcriptional reporter is dose-dependent. Co-transfection of CDK11 plasmid (20 ng) along with the transcriptional reporter resulted in ~2.7-fold activation, while increasing the amount of CDK11 to 40 ng lead to ~3.9-fold activation in U-2OS cells. Meanwhile, CDK11 siRNA (10 nM) decreased AR reporter activity by approximately 45% and a 20 nM dose of CDK11 siRNA decreased it further by 57%. In Fig. 3B, KHOS cells show similar results to U-2OS cells. In the presence of 20 ng or 40 ng of CDK11 plasmid transfection, AR reporter was activated by 2.5-fold and 3.4-fold respectively. AR reporter response was inhibited by nearly 36% and 53% when 10 nM or 20 nM of CDK11 siRNA was transfected. These data suggest that the transcription of AR is a downstream target of CDK11.

### Knockdown AR expression by siRNA inhibits osteosarcoma cell growth and viability

**Figure 4:**
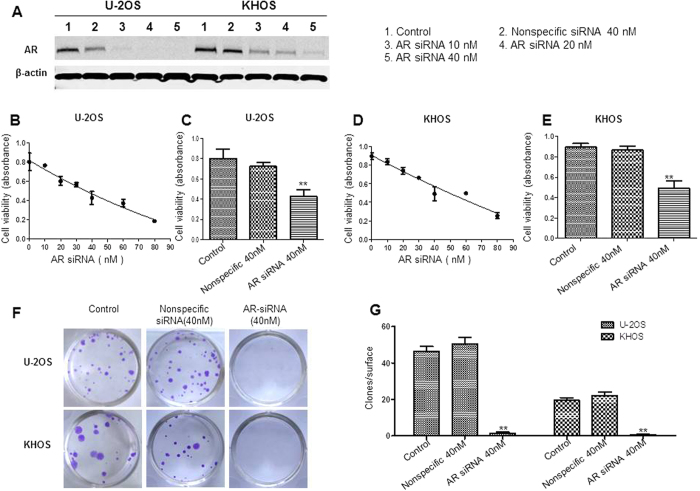
Effects of AR siRNA in osteosarcoma cell lines. (**A**) Expressions of AR in osteosarcoma cell lines with AR siRNA. (**B,C**) AR siRNA inhibits viability in osteosarcoma cell line U-2OS. The relative sensitivity of each line with AR siRNA was determined by MTT. (**D,E**) AR siRNA decreases cell viability in osteosarcoma cell line KHOS. (**F,G**) AR siRNA inhibits colony formation units in osteosarcoma cell line U-2OS and KHOS. *P < 0.05, **P < 0.01 (compared with control cells).

To characterize the functional role of AR in osteosarcoma cell growth and viability, AR siRNA was transfected in U-2OS and KHOS cell lines. Cells without any treatments or cells transfected with non-specific siRNA were used as control. Western blot analysis confirmed low-expression of AR in osteosarcoma cells transfected with AR siRNA and showed the AR expression closely correlated with cell viability (Fig. 4A). Both in U-2OS and KHOS cells, AR siRNA led to significant reduction of cell viability. Depletion of AR by siRNA resulted in cell death, which was not observed in the nonspecific siRNA transfection (Fig. 4B–E). The formation of CFU were assessed as indicator of osteosarcoma viability. The results of AR siRNA (40 nM) were compared with those obtained by nonspecific siRNA (40 nM) or blank control. We found that AR siRNA significantly inhibited CFU formation after 10 days of culture as compared to nonspecific siRNA or blank control (Fig. 4F,G). Meanwhile, we checked whether AR siRNA transfection could affect the migration of osteosarcoma cells in a wound-healing assay. Wounds in all three groups nearly recovered after 24 hours. However, this assay showed no significant differences among blank control, non-specific siRNA and AR siRNA groups neither at 8 hours nor 24 hours (Supplemental Figure S1). These data suggest that AR mediates osteosarcoma cell growth and viability, not migration, and thus likely contributes to osteosarcoma formation.

### AR inhibitor bicalutamide inhibits osteosarcoma cell viability and spheroid formation in 3D culture

**Figure 5:**
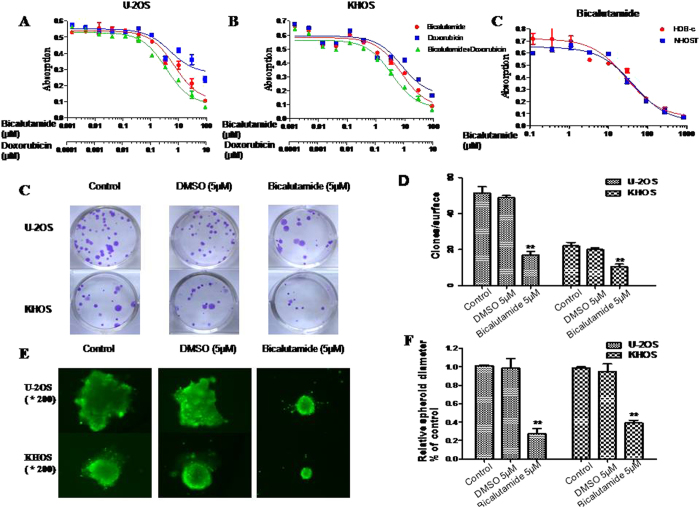
Effects of AR inhibitor bicalutamide in osteosarcoma cell lines. (**A,B**) Bicalutamide inhibits osteosarcoma cell viability. Cells were treated with bicalutamide or doxorubicin or the combination at the indicated concentrations. The relative sensitivity of each line was determined by MTT. (**C,D**) Bicalutamide inhibits colony formation units in osteosarcoma cell line U-2OS and KHOS. (**E,F**) Bicalutamide suppresses sphere formation of U-2OS and KHOS in three-dimensional culture. Spheroids formation of different cells after 7-day culture and the relative diameters compared with untreated cells. The assay was conducted in duplicate. *P < 0.05, **P < 0.01 (compared with control cells).

Bicalutamide is a synthetic, non-steroidal, AR antagonist used in the treatment of several cancers^25^,^26^,^27^. We tested the effect of this AR inhibitor, bicalutamide, on osteosarcoma cells. Doxorubicin is an anthracycline antitumor antibiotic and is commonly used to treat many cancers, including hematological malignancies, several types of carcinomas and soft tissue sarcomas^28^. The results of MTT assays demonstrated that bicalutamide significantly inhibited osteosarcoma cell viability (Fig. 5A–C). The IC50 of bicalutamide in KHOS was 7.475 μM and 7.121 μM in U-2OS. Compared with the effect of bicalutamide on osteosarcoma cells, osteoblast cells showed higher values of IC50 (HOB-c, 28.54 μM; NHOST, 42.32 μM). Further, the IC50 of doxorubicin in KHOS was 0.879 μM and 1.011 μM in U-2OS. The IC50 of the combined treatment in KHOS was 0.394 μM and 0.399 μM in U-2OS. This suggests that bicalutamide and doxorubicin synergistically inhibit osteosarcoma cell lines viability. Similar to the MTT assay result, the CFU assay showed that bicalutamide significantly inhibits osteosarcoma cell growth as well (Fig. 5C and D). Furthermore, in order to mimic the *in vivo* environment, a 3D cell culture was set-up. After 7 days of 3D growth in suspension, the diameter of spheroids formed by bicalutamide was smaller than DMSO or blank controls (Fig. 5E and F). Thereby, the results suggest that AR inhibitor bicalutamide significantly depressed the growth and progression of osteosarcoma cells. Bicalutamide has the potential to be used in osteosarcoma therapy, as it has synergistic effects with doxorubicin and causes greater growth inhibition of osteosarcoma than that of osteoblast cells.

## Discussion

In this study, we identified AR as a potential key player in CDK11 signaling in osteosarcoma. We utilized gene expression profiling of osteosarcoma cell lines with reduced CDK11 levels and reconstructed a gene network centered on CDK11. AR was shown to be key in the resulting network. Experiments performed here confirmed a role of AR signaling as it relates to CDK11 expression. We present here a first demonstration of the connection between CDK11 and AR in osteosarcoma.

It has been reported that AR is a central regulator of tumor progression in several types of hormone-associated cancers including prostate and breast cancers^29^,^30^,^31^,^32^. However, little is known about AR involvement in osteosarcoma. The significance of targeting AR signaling as a putative therapeutic in osteosarcoma is unknown. Based on the hypothesis generated by gene network reconstruction, we performed experiments to evaluate the relationship between CDK11 and AR in osteosarcoma. Both CDK11 and AR shared the same expression pattern in osteosarcoma cell lines and tumor tissues. When CDK11 expression was gradually reduced via siRNA knockdown, AR expression decreased accordingly. Moreover, there was a close relationship between CDK11 and AR staining patterns in osteosarcoma TMA representing various types of osteosarcoma. Both CDK11 and AR expression showed adverse predictive value for osteosarcoma patients in disease free survival rates. Consistent with these results, while this manuscript was in preparation, another study also demonstrated that AR expression is abundant osteosarcoma and AR is an independent prognostic indicator of overall survival and relapse-free survival in patients^33^. The prognostic value of AR has also been reported in prostate cancer. Correlations between AR expression and clinicopathologic variables were analyzed in 640 prostate cancer patients. High levels of AR are independently associated with increased viability, and are predictive of decreased biochemical recurrence-free survival^34^. These data imply that both CDK11 and AR may be potential prognostic factors predicting survival and outcome for patients with osteosarcoma, but larger validation studies are necessary. In addition, the transcriptional luciferase reporter assay suggests that CDK11 increases the transcriptional activation of the AR gene in a dose dependent manner. Another member of the CDK family, CDK6, also stimulates AR transcriptional activity in prostate cancer cells^35^. Expression of CDK6 markedly enhanced activation of the luciferase reporter in the presence of the AR in PC3 human prostate cancer cells. Meanwhile, no significant effects on AR activity were found with other members of CDK family, including CDK1, CDK2, and CDK4. Our data hints to a mechanism of CDK11 that is similar to CDK6 in regulation of AR gene transcription. CDK6 and CDK11 are distant homologs. Further, the p58 isomer of CDK11 cyclin D3 complex was shown to be involved in the repression of AR transcriptional activity^24^,^36^. However, no expression of p58 isomer of CDK11 in osteosarcoma cell lines was detected in our study. The detailed mechanism of AR regulation by CDK11 needs further investigation. The transcription factor FOXA1 binds directly to the AR promoter. FOXA1 has been shown to be involved in AR regulation in both breast and prostate cells^37^. It would be interesting to see if CDK11 regulation of AR is linked to FOXA1.

In humans, androgen secretion is regulated by gender and age. In men, androgens play a key role in the development of the testis and prostate and secondary sexual characteristics including bone mass increase^38^. Starting at puberty, the level of androgens in boys are over 10 times that of girls, although androgens exist in both sexes^39^. However, androgens levels decrease rapidly with age^40^. As osteosarcomas tend to occur most commonly in young people with a higher incidence in males, we then evaluated the relationship between AR expression and gender or age. We divided the patients into two groups: 24 years old or younger in one group, and older than 24 in the second. However, no significant differences of AR expression were found either in gender or age. It should be pointed out that, although not statistically significant, the patients less than 24 years old seemed to have a tendency towards higher AR expression than older adults (*P* = 0.137). As some factors such as osteosarcoma subtypes and stage of disease might also affect the above results, larger numbers of osteosarcoma patients in other populations should be further studied.

Prostate cancer depends on AR signaling and has been well studied within the context of this cancer. In fact, metastatic prostate cancer cells are able to maintain AR signaling throughout this cancer development. The role of AR in osteosarcoma is not understood. In this study, by differential gene expression we found several genes that might be the AR downstream target genes in osteosarcoma, such as cyclin G2, CDC20, Caspase-8, JNK. Most of those genes have been reported to be involved in AR signaling and cancer prostate cancer. Those data suggest that, AR signaling might also play an important role in osteosarcoma. Androgen-deprivation therapy for prostate cancer was described several decades ago and remains common practice^41^. Recently, attention to the AR in breast cancer research increased, with results supporting AR inhibitor as a treatment for AR^+^ breast cancer^42^. In hepatocellular carcinoma and prostate cancer, AR is widely expressed as well and plays a similar inhibition role in cancer cell growth^35^,^43^. To determine the role of AR in osteosarcoma, we evaluated the effects of AR siRNA and AR specific inhibitor (bicalutamide) in osteosarcoma cell lines. Both AR siRNA and bicalutamide significantly suppressed osteosarcoma cell viability and growth, but not migration. Our results are in line with a recent study showing knockdown of AR inhibited the viability of osteosarcoma cells^33^. The important role of AR in skeletal growth has already been well described, which may contribute to its oncogenic role in osteosarcoma. Bone homeostasis is most important during times of bone accrual and bone remodeling. Androgens act through the AR in mineralizing osteoblasts and maintaining bone homeostasis. Bone resorption, bone matrix synthesis and mineralization are regulated through the AR^44^. An association between the peak of childhood skeletal growth and the top incidence of osteosarcoma in adolescent has been documented^45^. Hormonal activity, specifically androgens, is a primary stimulus of skeletal growth^46^. As osteoblasts are commonly involved in the osteosarcoma pathological process, the oncogenic role of AR may be partly carried out by regulating bone homeostasis.

Prior to the approval of enzalutamide (a non-steroidal anti-androgen with improved effectiveness), bicalutamide was considered as the standard-of-care anti-androgen for the treatment of prostate cancer. Relative to the non-steroidal anti-androgens flutamide and nilutamide, bicalutamide has the highest affinity for the AR^47^ and is well-tolerated^48^. Further, bicalutamide is still being studied in other cancers, including breast^49^ and ovarian cancer^11^. The IC50 of bicalutamide in KHOS or U-2OS was less than 10 μM, which is consistent with the record in the Genomics of Drug Sensitivity in Cancer database (http://www.cancerrxgene.org/). Bicalutamide and doxorubicin suggested synergistic effects in the inhibition of viability in osteosarcoma cell lines. Bicalutamide with tamoxifen have a synergistic inhibitory influence on prostate cancer growth and insulin-like growth factor 1 expression in prostate cancer patients^50^. In addition, osteosarcoma patients with high expression of AR showed good response to preoperative chemotherapy. These findings suggest a possible application of AR assessment in osteosarcoma treatment. We used 3D growth in suspension was used in this study as a closer setting to *in vivo* conditions. After 7 days of culture in 3D environment, the diameter of spheroids formed was significantly inhibited by bicalutamide compared with blank or DMSO control groups. Thereby, AR inhibitors may be effective in osteosarcoma treatment both *in vivo* and *in vitro*. Given that multiple agents targeting AR are currently in preclinical or clinical development, the results from this study may lead to another tool in the chemotherapy for osteosarcoma clinical trials.

In conclusion, the identification of a link between CDK11 and AR transcriptional regulation provides a means for prioritizing key targets in potential osteosarcoma therapeutic interventions. High expression of AR has been shown in osteosarcoma cell lines and tissues, and its inhibition attenuates cell growth and viability. A correlation between AR and survival of osteosarcoma patients implicates the high expression of AR as a potential biomarker for prognosis. We speculate that a CDK11-AR relationship will be particularly useful in targeting oncogenic pathways utilizing a combinatorial therapeutic strategy in the treatment of osteosarcoma.

## Additional Information

**How to cite this article**: Liao, Y. *et al*. Androgen receptor is a potential novel prognostic marker and oncogenic target in osteosarcoma with dependence on CDK11. *Sci. Rep.* **7**, 43941; doi: 10.1038/srep43941 (2017).

**Publisher's note:** Springer Nature remains neutral with regard to jurisdictional claims in published maps and institutional affiliations.

## Supplementary Material

Supplementary Information

## Figures and Tables

**Table 1:** The relationship between AR expression and clinicopathological features of osteosarcoma.

	Clinicopathological features	No. of cases(%)	AR expression High (n)	AR expression Low (n)	*P* value
All patients		67 (100)			
Age, y	≤24	35 (52.2)	18 (51.4)	17 (48.6)	0.137
	>24	32 (47.8)	10 (31.3)	22 (68.7)	
Gender	Male	40 (62.1)	16 (40.0)	24 (60.0)	0.803
	Female	27 (37.9)	12 (44.4)	15 (55.6)	
Grade	Grade 1	9 (13.4)	3 (3.3)	6 (6.7)	0.0662
	Grade 2	31 (46.3)	14 (45.2)	17 (54.8)	
	Grade 3	27 (40.3)	19 (70.4)	8 (29.6)	
Metastasis	Absent	22 (32.8)	9 (40.9)	13 (59.1)	0.566
	Present	45 (67.2)	19 (42.2)	26 (57.8)	
Recurrence	Absent	47 (70.1)	20 (42.6)	27 (57.4)	0.533
	Present	20 (29.9)	8 (40.0)	12 (60.0)	
Response to preoperative chemotherapy	Good (Necrosis over 90%)	9 (12.1)	6 (62.5)	3 (37.5)	0.047
	Poor	37 (54.6)	10 (41.7)	27 (58.3)	
	N/A	21 (33.3)	12 (59.1)	9 (40.9)	
5-Year Survival (%)	Absent		10	23	0.084
	Present		18	16	
AR expression	High		28 (41.8)		
	Low			39 (58.2)	
