# Author Correction: Androgen receptor is a potential novel prognostic marker and oncogenic target in osteosarcoma with dependence on CDK11

**DOI:** 10.1038/s41598-018-30644-x

**Published:** 2018-08-09

**Authors:** Yunfei Liao, Slim Sassi, Stefan Halvorsen, Yong Feng, Jacson Shen, Yan Gao, Gregory Cote, Edwin Choy, David Harmon, Henry Mankin, Francis Hornicek, Zhenfeng Duan

**Affiliations:** 1 Sarcoma Biology Laboratory, Department of Orthopaedic Surgery, Massachusetts General Hospital and Harvard Medical School, 55 Fruit Street, Jackson 1115, Boston, Massachusetts, 02114 USA; 2 Department of Endocrinology, Wuhan Union Hospital, Tongji Medical College, Huazhong University of Science and Technology, 1277 Jie Fang Avenue, Wuhan, 430022 China; 3 Center for Computational and Integrative Biology (CCIB), Massachusetts General Hospital, Boston, Massachusetts, 02139 USA; 4 Department of Orthopaedic Surgery, Wuhan Union Hospital, Tongji Medical College, Huazhong University of Science and Technology, 1277 Jie Fang Avenue, Wuhan, 430022 China; 5 Division of Hematology and Oncology, Massachusetts General Hospital and Harvard Medical School, Boston, Massachusetts, 02114 USA

Correction to: *Scientific Reports* 10.1038/srep43941, published online 06 March 2017

In this Article, the U-20S Bicalutamide image in Figure 5C is a duplication of the KHOS control image in Figure 4F. The correct Figure 5 appears below as Figure 1.

**Figure 1:**
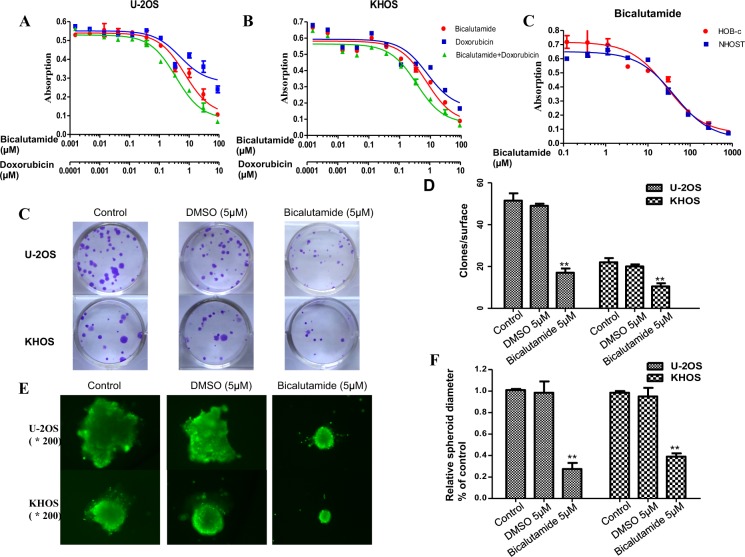
Effects of AR inhibitor bicalutamide in osteosarcoma cell lines. (**A**,**B**) Bicalutamide inhibits osteosarcoma cell viability. Cells were treated with bicalutamide or doxorubicin or the combination at the indicated concentrations. The relative sensitivity of each line was determined by MTT. (**C**,**D**) Bicalutamide inhibits colony formation units in osteosarcoma cell line U-2OS and KHOS. (**E**,**F**) Bicalutamide suppresses sphere formation of U-2OS and KHOS in three-dimensional culture. Spheroids formation of different cells after 7-day culture and the relative diameters compared with untreated cells. The assay was conducted in duplicate. *P < 0.05, **P < 0.01 (compared with control cells).

