# Author Correction: Androgen receptor is a potential novel prognostic marker and oncogenic target in osteosarcoma with dependence on CDK11

**DOI:** 10.1038/s41598-024-51815-z

**Published:** 2024-01-22

**Authors:** Yunfei Liao, Slim Sassi, Stefan Halvorsen, Yong Feng, Jacson Shen, Yan Gao, Gregory Cote, Edwin Choy, David Harmon, Henry Mankin, Francis Hornicek, Zhenfeng Duan

**Affiliations:** 1 Sarcoma Biology Laboratory, Department of Orthopaedic Surgery, Massachusetts General Hospital and Harvard Medical School, 55 Fruit Street, Jackson 1115, Boston, MA USA; 2 Department of Endocrinology, Wuhan Union Hospital, Tongji Medical College, Huazhong University of Science and Technology, 1277 Jie Fang Avenue, Wuhan, 430022 China; 3 Center for Computational and Integrative Biology (CCIB), Massachusetts General Hospital, Boston, MA 02139 USA; 4 Department of Orthopaedic Surgery, Wuhan Union Hospital, Tongji Medical College, Huazhong University of Science and Technology, 1277 Jie Fang Avenue, Wuhan, 430022 China; 5 Division of Hematology and Oncology, Massachusetts General Hospital and Harvard Medical School, Boston, MA 02114 USA

Correction to: *Scientific Reports* 10.1038/srep43941, published online 06 March 2017

The original Article contains errors. Due to a mistake during figure assembly, the western blot panels in Figure 2C were duplicated from ^1^. The corrected Figure 2 appears below as Figure 1.

**Figure 1:**
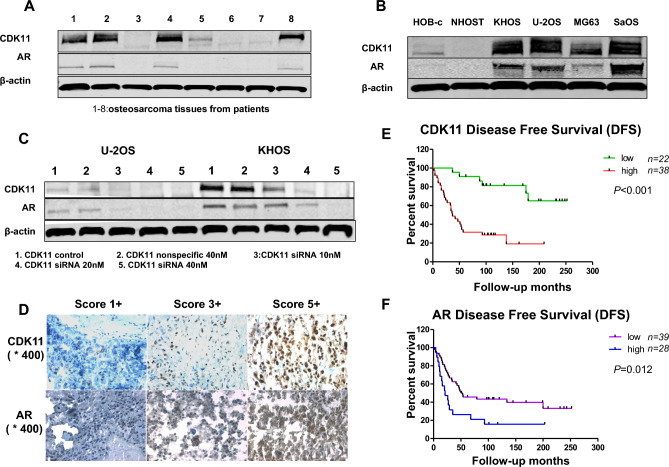
(**A**) Expressions of CDK11 and AR in osteosarcoma tissues. (**B**) Expressions of CDK11 and AR in osteosarcoma cell lines and normal osteoblast cell lines. (**C**) Expression of AR in osteosarcoma with CDK11 siRNA. (**D**) Representative images of different immunohistochemical staining intensities of AR and CDK11 are shown in osteosarcoma tissues. The percentage of cells showing positive nuclear staining for AR and CDK11 was calculated by reviewing the entire spot. On the basis of the percentage of cells with positive nuclear staining, the staining patterns were categorized into 6 groups: 0, no nuclear staining; 1, 1+, <10% of cells stained positive; 2, 2+, 10% to 25% positive cells; 3, 3+, 26% to 50% positive cells; 4, 4+, 51% to 75% positive cells; and 5, 5+, >75% positive cells (Original magnification, ×400). (**E**) Kaplan–Meier survival curve of patients with osteosarcoma were subgrouped as either CDK11 low staining (staining ≤2) or high staining (staining ≥3). (**F**) Kaplan–Meier disease free survival curve of patients with osteosarcoma were subgrouped as either AR low staining (AR staining ≤2) or high staining (AR staining ≥3).
