# Supplementary material for: Androgen receptor is a potential novel prognostic marker and oncogenic target in osteosarcoma with dependence on CDK11
Source: Sci Rep. 2017 Mar 6;7:43941. doi: 10.1038/srep43941 (PMC5338289; doi:10.1038/srep43941)
Supplement: Supplementary Information [file srep43941-s1.pdf]

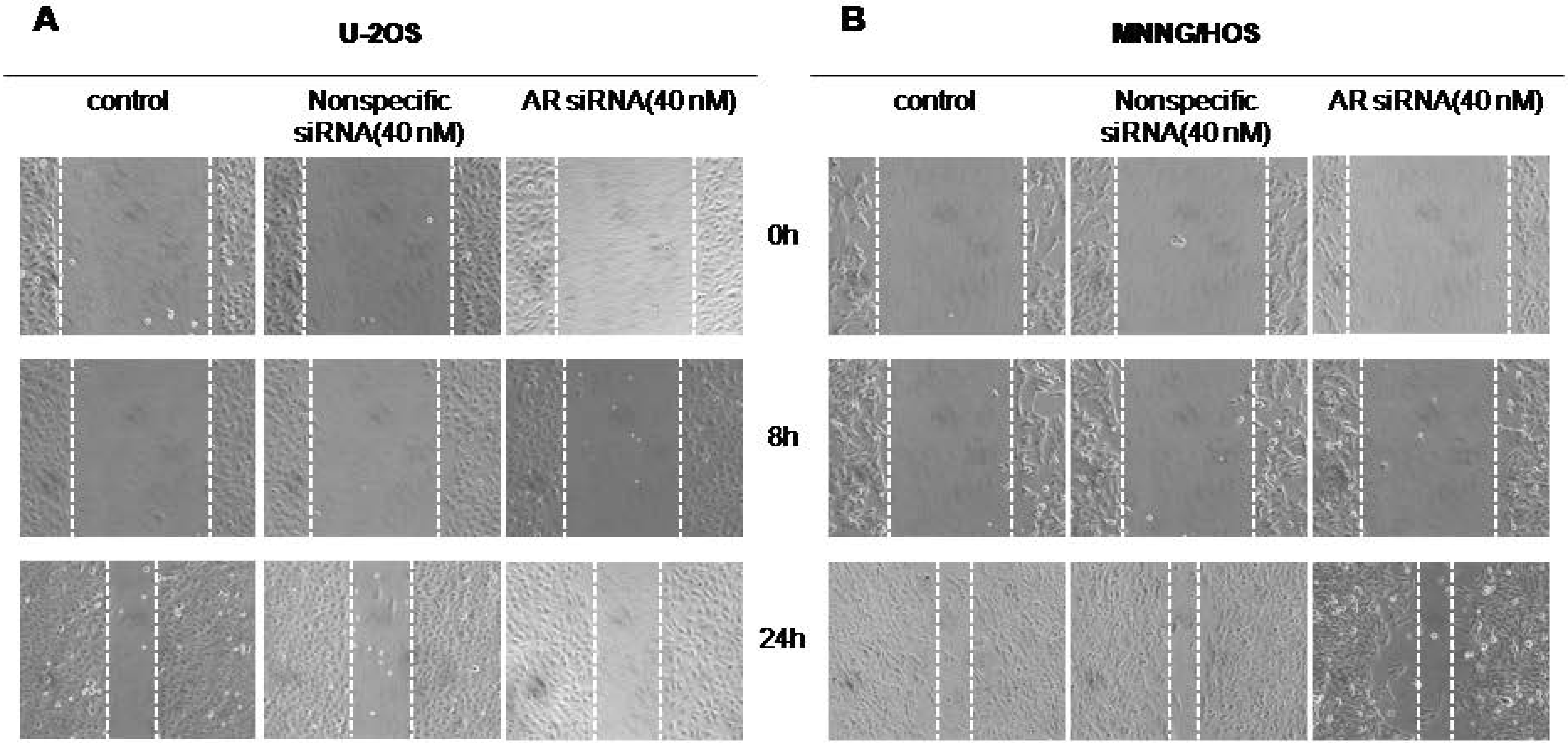
A
U-2OS
control
Nonspecific siRNA(40 nM)
AR siRNA(40 nM)
0h
8h
24h
B
MNNG/HOS
control
Nonspecific siRNA(40 nM)
AR siRNA(40 nM)

Androgen receptor is a potential novel prognostic marker and oncogenic target in osteosarcoma with dependence on CDK11

Yunfei Liao[1,2], Slim Sassi[1,3], Stefan Halvorsen[3], Yong Feng[1,4], Jacson Shen[1], Yan Gao[1], Gregory Cote[5], Edwin Choy[5], David Harmon[5], Henry Mankin[1], Francis Hornicek[1], and Zhenfeng Duan[1]

AUTHOR AFFILIATIONS:

[1] Sarcoma Biology Laboratory, Department of Orthopaedic Surgery, Massachusetts General Hospital and Harvard Medical School, 55 Fruit Street, Jackson 1115, Boston, Massachusetts 02114

[2] Department of Endocrinology, Wuhan Union Hospital, Tongji Medical College, Huazhong University of Science and Technology, 1277 Jie Fang Avenue, Wuhan, China, 430022

[3] Center for Computational and Integrative Biology (CCIB), Massachusetts General Hospital, Boston, Massachusetts 02139

[4] Department of Orthopaedic Surgery, Wuhan Union Hospital, Tongji Medical College, Huazhong University of Science and Technology, 1277 Jie Fang Avenue, Wuhan, China, 430022

[5] Division of Hematology and Oncology, Massachusetts General Hospital and Harvard Medical School, Boston, Massachusetts 02114

Correspondence: Zhenfeng Duan, Sarcoma Biology Laboratory, Department of Orthopaedic Surgery, Massachusetts General Hospital and Harvard Medical School, 55 Fruit Street, Jackson 1115, Boston, Massachusetts 02114. Tel: 617-724-3144; Fax: 617-726-3883; *E-mail address*: zduan@mgh.harvard.edu

Supplemental Figure 1. AR siRNA does not show the effects of the migratory in osteosarcoma cell lines. (A) Micrographs of osteosarcoma U-2OS cells at 0, 8, and 24 hours after wounding. (B) Micrographs of osteosarcoma MNNG/HOS cells at 0, 8, and 24 hours after wounding. Micrographs of osteosarcoma cells transfected with AR siRNA or nonspecific siRNA. The invading cells were stained with hematoxylin. $^{*}P < 0.05$, $^{**}P < 0.01$ (compared with control cells).

Supplemental table 1

Gene_Symbol	KHOS_CDK11_siRNA	KHOS_control	KHOS_non-specific_siRNA	KHOS_Fold_Activation CDK11 vs. non-specific	U2OS_CDK11_siRNA	U2OS_control	U2OS_non-specific_siRNA	U2OS_Fold_Activation CDK11 vs. non-specific	Average_U2OS_KHOS_Activation
**GNAS-AS1**	2.34	5.42	5.70	**0.41**	5.70	6.83	6.89	**0.83**	**0.62**
**ACTR3**	5.02	7.07	6.83	**0.73**	2.91	4.08	4.74	**0.61**	**0.67**
**CDK11**	6.45	8.35	8.55	**0.75**	4.97	7.81	7.61	**0.65**	**0.70**
**TMEM19**	5.22	7.76	7.51	**0.69**	4.84	7.45	6.80	**0.71**	**0.70**
**LOC100505761**	2.87	5.43	5.11	**0.56**	6.69	8.09	7.91	**0.85**	**0.70**
**TBRG1**	5.16	6.43	6.67	**0.77**	2.87	4.40	4.39	**0.65**	**0.71**
**CBX5**	3.74	5.53	5.53	**0.68**	5.56	6.26	6.99	**0.80**	**0.74**
**UBE2K**	5.98	9.42	9.14	**0.65**	6.21	8.49	7.50	**0.83**	**0.74**
**RPRD1A**	7.59	9.21	8.98	**0.85**	4.58	6.97	7.16	**0.64**	**0.74**
**USP13**	7.06	9.17	8.97	**0.79**	4.96	7.91	6.92	**0.72**	**0.75**
**B9D1**	4.75	6.84	7.23	**0.66**	6.18	7.06	7.28	**0.85**	**0.75**
**CCDC15**	5.77	7.33	7.14	**0.81**	3.55	4.82	5.01	**0.71**	**0.76**
**TMEM68**	6.91	9.16	8.80	**0.78**	5.25	6.96	6.82	**0.77**	**0.78**
**SEC22C**	5.17	6.57	7.02	**0.74**	6.78	8.04	8.16	**0.83**	**0.78**
**HOTAIRM1**	7.79	9.08	9.30	**0.84**	5.84	7.28	7.68	**0.76**	**0.80**
**LEO1**	8.87	10.19	10.28	**0.86**	6.07	8.12	8.13	**0.75**	**0.81**
**CDC7**	7.04	9.58	9.13	**0.77**	6.93	8.42	8.19	**0.85**	**0.81**
**USP3**	5.95	7.28	7.39	**0.81**	5.48	6.57	6.67	**0.82**	**0.81**
**CDC27**	5.75	7.31	7.12	**0.81**	5.91	7.76	7.13	**0.83**	**0.82**
**ZFP62**	7.98	10.13	10.04	**0.79**	8.16	10.12	9.66	**0.84**	**0.82**
**GSTCD**	6.93	8.78	8.82	**0.79**	7.47	8.54	8.57	**0.87**	**0.83**
**RBFOX2**	7.74	8.83	9.20	**0.84**	7.30	9.00	8.82	**0.83**	**0.83**
**CBFB**	9.59	11.64	11.74	**0.82**	9.22	10.95	10.77	**0.86**	**0.84**
**NKAP**	7.68	9.00	9.07	**0.85**	6.39	7.79	7.63	**0.84**	**0.84**
**CTSZ**	7.25	8.49	8.62	**0.84**	7.16	8.86	8.50	**0.84**	**0.84**
**CCDC82**	7.07	8.48	8.60	**0.82**	7.70	8.58	8.87	**0.87**	**0.85**
**MAP2K4**	8.18	9.87	9.53	**0.86**	7.00	8.99	8.42	**0.83**	**0.85**
**MCM3**	9.22	10.87	10.81	**0.85**	9.39	11.46	11.05	**0.85**	**0.85**
**HELLS**	7.96	9.65	9.36	**0.85**	5.40	6.10	4.13	**1.31**	**1.08**
**SMIM15**	12.76	12.81	11.22	**1.14**	13.25	13.16	11.96	**1.11**	**1.12**
**LCORL**	9.43	8.86	7.94	**1.19**	8.60	8.53	7.49	**1.15**	**1.17**
**CSGALNACT2**	11.57	10.92	9.51	**1.22**	11.19	10.84	9.84	**1.14**	**1.18**
**PTX3**	9.78	10.41	8.29	**1.18**	9.81	9.90	8.27	**1.19**	**1.18**
**TM2D1**	8.27	6.90	6.78	**1.22**	7.73	6.31	6.35	**1.22**	**1.22**
**PTHLH**	9.80	8.21	7.98	**1.23**	7.18	5.49	5.78	**1.24**	**1.23**
**ZNF367**	9.25	8.94	7.49	**1.23**	9.51	9.16	7.69	**1.24**	**1.24**
**SCUBE3**	7.48	7.20	6.10	**1.23**	5.68	4.98	4.51	**1.26**	**1.24**
**NHLRC3**	8.51	7.50	6.42	**1.33**	8.45	7.90	7.23	**1.17**	**1.25**
**CDADC1**	6.97	6.33	5.58	**1.25**	6.05	5.83	4.82	**1.25**	**1.25**
**RAB11FIP1**	6.41	5.67	4.88	**1.32**	8.15	7.99	6.73	**1.21**	**1.26**
**NACA**	8.48	6.30	6.34	**1.34**	7.71	6.40	6.29	**1.23**	**1.28**
**NEDD4**	10.67	10.14	8.79	**1.21**	6.53	6.83	4.76	**1.37**	**1.29**
**FAM73A**	8.23	6.98	6.09	**1.35**	6.67	5.02	5.24	**1.27**	**1.31**
**SLC16A6**	10.24	7.01	7.66	**1.34**	5.04	4.07	3.66	**1.38**	**1.36**
**SGMS2**	5.37	4.75	3.40	**1.58**	8.63	7.96	7.39	**1.17**	**1.37**
**DSC2**	6.05	5.58	4.32	**1.40**	7.39	6.71	5.31	**1.39**	**1.40**
**STC1**	11.03	9.29	9.66	**1.14**	7.18	4.42	4.07	**1.76**	**1.45**
**FAHD2CP**	3.77	4.18	2.23	**1.69**	6.87	6.53	5.61	**1.22**	**1.46**

Supplemental Table 2. Multivariate Survival Analysis

Variable	5-Year survival			5-Year disease-free survival		
	Hazard Ratio	95 % CI	*P*	Hazard Ratio	95 % CI	*P*
Metastasis	2.877	1.840 - 4.262	0.013	3.15	2.076-4.963	0.009
AR expression	1.836	1.259 - 2.738	0.128	1.961	1.465 - 2.798	0.067
Response to preoperative chemotherapy	2.095	1.446-3.204	0.084	2.332	1.811-3.897	0.059
